# Phages and Enzybiotics in Food Biopreservation

**DOI:** 10.3390/molecules26175138

**Published:** 2021-08-25

**Authors:** José Ramos-Vivas, María Elexpuru-Zabaleta, María Luisa Samano, Alina Pascual Barrera, Tamara Y. Forbes-Hernández, Francesca Giampieri, Maurizio Battino

**Affiliations:** 1 Research Group on Foods, Nutritional Biochemistry and Health, Universidad Europea del Atlántico, 39011 Santander, Spain; jose.ramos@uneatlantico.es (J.R.-V.); maria.elexpuru@uneatlantico.es (M.E.-Z.); marialuisa.samano@unini.edu.mx (M.L.S.); 2 Department of Project Management, Universidad Internacional Iberoamericana, Campeche 24560, Mexico; alina.pascual@unini.edu.mx; 3 Department of Analytical and Food Chemistry, CITACA, CACTI, University of Vigo, 36310 Vigo, Spain; tforbes@uvigo.es; 4 Department of Clinical Sciences, Polytechnic University of Marche, 60131 Ancona, Italy; 5 Department of Biochemistry, Faculty of Sciences, King Abdulaziz University, Jeddah 21589, Saudi Arabia; 6 International Research Center for Food Nutrition and Safety, Jiangsu University, Zhenjiang 212013, China

**Keywords:** bacteriophage, endolysin, enzybiotics, biopreservation

## Abstract

Presently, biopreservation through protective bacterial cultures and their antimicrobial products or using antibacterial compounds derived from plants are proposed as feasible strategies to maintain the long shelf-life of products. Another emerging category of food biopreservatives are bacteriophages or their antibacterial enzymes called “phage lysins” or “enzybiotics”, which can be used directly as antibacterial agents due to their ability to act on the membranes of bacteria and destroy them. Bacteriophages are an alternative to antimicrobials in the fight against bacteria, mainly because they have a practically unique host range that gives them great specificity. In addition to their potential ability to specifically control strains of pathogenic bacteria, their use does not generate a negative environmental impact as in the case of antibiotics. Both phages and their enzymes can favor a reduction in antibiotic use, which is desirable given the alarming increase in resistance to antibiotics used not only in human medicine but also in veterinary medicine, agriculture, and in general all processes of manufacturing, preservation, and distribution of food. We present here an overview of the scientific background of phages and enzybiotics in the food industry, as well as food applications of these biopreservatives.

## 1. Introduction

Food preservation by suitable means is key in food safety and quality. There are several traditional and well-known physical preservation techniques such as refrigeration and pasteurization, but the modern industry is always looking for new procedures for food preservation to increase the product’s shelf-life by minimizing the loss of nutritional quality and organoleptic properties. Presently, some modern biopreservation techniques rely on naturally occurring microorganisms (i.e., lactic acid bacteria) and their metabolites. These food preservatives are mainly used to produce safer food for the consumer, preventing the action of pernicious microbes which can cause food deterioration or even toxicity and therefore be dangerous to human health.

Moreover, bacteria -including multidrug-resistant bacteria- can reach food at different points in the food supply chain, from farm to postharvest, and processing such as slaughtering, fermentation, packaging and storage [1,2,3,4,5].

As most natural foods are highly perishable, by extending their half-life we can also control their native microbiota for proper preservation, maintaining their safety and quality. As microorganisms produce a long list of molecules ranging from classic antibiotics to antibacterial enzymes, the control of indigenous populations in food can be achieved by adding these products directly. The paradigm of bacterial molecules used in the food industry as biopreservatives is Nisin, a bacteriocin produced by the Gram-positive bacterium *Lactococcus lactis*, one of the lactic acid bacteria most extensively used for the manufacture of dairy products [6]. Other well-known bacteriocins, such as Pediocin, Natamycin, Enterocin, and Leucocin [7], also have inhibitory properties against other microorganisms which makes them very interesting for use in the food industry. Some bacteria that produce these compounds have been used as probiotics. Current research on probiotics is quite promising and modern fashion trends push probiotics and bacteriocins from modulation of the gut microbiota toward a wide range of other health-promoting activities away from food, such as cancer treatment, skin health care, periodontal health, or allergies [8,9,10,11].

In addition, the use of bacteriocin producing strains or those that can compete against pathogens in the context of the food industry needs new approaches, mainly due to the increase in foodborne infections, the appearance of new production processes, the massive demand for food, and the changing consumer trends. Moreover, the extensive use of antibiotics against animal and human pathogens has also led to an increase in foodborne pathogens resistant to antibiotics, which makes the picture not reassuring at all [12,13,14].

Goodridge and Abedon published an article in 2003 where they proposed to use the terms “phage biocontrol” and “phage bioprocessing” to differentiate the application of bacteriophages in the farm or crops from their use in the food industry [15]. Several years later, Greer published a review of the control of foodborne bacteria using phages, including the effects of these microorganisms on food storage and preservation [16].

At that time, the excellent properties of endolysins to kill bacteria were already known, but their use to protect food from foodborne pathogens had not yet been effectively tested. One of the first murein hydrolases to be studied concerning food-related bacteria was that of the *Lactobacillus helveticus* bacteriophage 0303 [17]. This endolysin exhibited a broad spectrum of activity, killing different bacterial species such *Pediococcus acidilactici*, *Lactobacillus delbrueckii* subsp. *bulgaricus*, *Lactobacillus delbrueckii* subsp. *lactis*, *Lactobacillus acidophilus*, *Bacillus subtilis*, *Enterococcus faecium*, and several strains of *Lactobacillus helveticus*.

Problems of deterioration of the organoleptic properties have been described after physical treatments; also, consumers are increasingly demanding low-processed foods. One of the advantages of phages over the usual physical treatments is that phages do not modify any organoleptic properties of foods. Moreover, even with common treatments such as heat, team and UV light, a relatively high percentage of food products are lost due to subsequent microbial spoilage or microbial contamination; when food becomes contaminated, it will lead to food spoilage, and such food will no longer be fit for consumption.

Thanks to their ability to control or to inactivate spoilage and/or foodborne bacteria selectively, bacteriophages have great potential as food biopreservatives. Additionally, in terms of food biopreservation, enzybiotics are beginning to be increasingly studied in the field of food microbiology, taking advantage of the pull that in vitro successes have displayed against very important multidrug-resistant human and animal pathogens [18,19,20].

In this review, we discuss the use of phages and their lytic enzymes as a tool to eliminate or reduce spoilage bacteria and common foodborne bacterial pathogens.

## 2. Why Bacteriophages?

Bacteriophages are an alternative to antimicrobials in the fight against bacteria, mainly because they have a practically unique host range, which gives them great specificity. Apart from their selective activity, bacteriophages have been successfully tested to eliminate or weaken biofilms formed by different classes of both Gram-negative and Gram-positive pathogens in the food industry [21,22,23,24]. Biofilms are consortia of bacteria that persist on different surfaces or pipelines within the food industries that contaminate food at some point in the processing or packaging chain.

In addition to their potential ability to specifically control strains and biofilms of pathogenic bacteria, their use does not generate a negative environmental impact like in the case of antibiotics or disinfectants [25]. Other advantages of these viruses are: (i) safety—as they are not toxic to eukaryotic cells, (ii) the preservation of the organoleptic properties of food, and (iii) the control of multi-resistant bacteria since the tolerance of some strains to phages can often be overcome with the use of phage cocktails [14]. In addition, phages can be used in combination with antibiotics, bacteriocins, or even with probiotics.

The main limitations of bacteriophages as biopreservative tools in foods derive from the scarce knowledge of their genetics since the use of strains that may contain virulence factors, lysogeny, or antibiotic resistance genes is inadvisable. As an example, studies prior to this decade did not have the modern and inexpensive sequencing techniques that almost all laboratories can afford today. Furthermore, in some cases, it is necessary to use phage cocktails that are more difficult to characterize than individual strains. Additionally, we need to learn much more about their behavior within solid and liquid food matrices to optimize the amount of phage to be used in each case. The method of releasing phages on food is also important, since the phages must reach the largest number of bacteria possible so that they can effectively control them and reduce their number to safe values. In other words, phages and bacteria must be in contact with liquid but also with solid foods; moreover, as much bacterial contamination occurs initially at low numbers (a minimum bacterial density is a prerequisite) sometimes we must apply a large number of phages to those foods. Knowing the optimal number of viral particles (multiplicity of infection, MOI) to use for each food, as well as their infection kinetics in each food matrix, it is essential to understand how these phages are acting on their target pathogens [26,27,28,29,30,31,32]. Minimum host threshold requirement has been demonstrated for phages of different food pathogens [33,34]. As successful biopreservative agents, it is also important to consider phages’ stability in food matrices under different environmental conditions such as water activity, salinity, temperature, pH, osmotic shock, and light (visible and UV). According to several authors, phages have a remarkable stability in foods [35,36,37]. Phage propagation on a susceptible host, purification, and phage or cocktail formulation are very relevant parameters too.

In some studies, in which a high number of phages are used, the bacterial lysis ‘from without’ can occur because many viral particles bind to the bacterial surface, leading to the production of numerous holes in the cell wall [38,39]. All these concepts must be better studied and understood in order to apply phages to food pathogens.

Although the application of phages will continue, there is a phenomenon that must always be kept in mind, the emergence of phage-resistant strains. When infecting bacterial cells, phages already face a range of antiviral mechanisms (i.e., restriction modification systems/enzymes), and they have evolved multiple tactics to avoid these mechanisms. In this co-evolution between bacteria and phages, most authors agree that phages can effectively raise a counter-resistance. Therefore, finding a new phage that can infect a bacterium will always be easier than finding an entirely novel family of antibiotics.

We do not know much about how often these resistant variants of phages used in the food industry appear, as few publications include assays to study this phenomenon. It is likely that researchers prioritize the study of efficacy over safety. Moreover, multidrug resistance, where a bacterium has obtained resistance mechanisms against several different families of antibiotics, is increasingly common, but this phenomenon does not occur when phages are used. Additionally, many studies suggest that phage combinations can be optimized to limit the emergence and persistence of resistance, therefore promoting the long-term usefulness of phage therapy. With regards to this issue, enzybiotics offer the advantage that they do not generate resistance because they act on essential targets for the bacteria’s viability, so, it is difficult for bacteria to modify them.

The other most important issue in addition to the development of phage-resistant strains is phage spread. As bacteriophages applied to food can be easily transferred between facilities in the food industry, we must pay particular attention to the number of phages used, and above all, to how they are applied to food. An undesirable effect would be the inactivation of starter cultures that initiate the fermentation processes. Despite the narrow spectrum of a specific phage, the problem of the phages spread within the food industries is real because it is not convenient; for example, to collaterally eliminate some species of lactic acid bacteria that confer characteristic properties to the products in which they are present [40].

As with isolated phages, phage cocktails can be used directly on food or surfaces and food handling tools in chain processing plants. Another advantage of phage cocktails is that they can be modified quickly and conveniently to deal with specific strains that may appear in a particular food manufacturing facility [41]. No articles were reviewed here where more than three bacteriophages or cocktails containing undefined strains were used because in the last few years there have been excellent reviews on that scope [26,41,42,43]. Moreover, Theuretzbacher’s recent article in the currently available weaponry against superbugs indicates that more than 20 different bacteriophage-based products have been approved for the control of pathogenic bacteria related to the food industries or direct food contamination [44].

Our review of approximately 100 bacteriophages indicates that three families (*Myoviridae*, *Siphoviridae*, and *Podoviridae*) account for the majority of virulent phages for the most common food-borne pathogen species. Much work has focused on the biocontrol or biopreservation of foods with six of the most important food-borne pathogens: *E. coli* (mainly serotype 015:H7), *Listeria monocytogenes*, *Staphylococcus aureus*, *Clostridium* spp., *Campilobacter jejuni*, and *Salmonella* spp., (Table 1). In addition to those six important food pathogens, phages against many other bacteria capable of causing foodborne infections should begin to be studied. This would allow us to identify not only new phages but also interesting enzybiotics.

According to the articles analyzed, the phages of the family *Myoviridae* were preferentially used to control *E. coli*. Other important food pathogens such as *C. jejuni*, *Salmonella*, *L. monocytogenes*, and *S. aureus* were controlled by *Siphoviridae* and *Myoviridae*. The analyzed studies showed that the *Podoviridae* family can infect all these species, but fewer phage strains of this family have been found to control bacteria in the different foods tested. Comparative genomics and morphological observation by transmission electron microscopy revealed that the phage LPSEYT, able to infect *Salmonella*, represents a new genus within the *Myoviridae* family [42]. This last example shows that if we go a little deeper into the genomic characterization of the isolated strains, we will be able to advance in the knowledge of the taxonomy of phages. Most of the phages used to control these pathogen species in food were isolated from wastewater, sewage, or other environmental samples; but many have also been isolated from different foods. One phage strain (EcpYZU01) of the *Corticoviridae* family was isolated from sewage samples and tested against *Enterobacter cloacae* in cucumber juice [43]. Finally, a phage (LPST94) from the *Ackermannviridae* family isolated from water was effective against *Salmonella* in foods [108,109]. This newly assigned family was recently added to the list of the International Committee on Taxonomy of Viruses ICTV catalog. The isolation of phages from sewage and water samples is common due to their abundance in these ecosystems. However, Scattolini et al., pointed out that the search and characterization of phages isolated in the same foods in which the pathogens can hide could be a good way “to integrate this control measure in an innovative, cost-effective, safe and environmentally friendly way” [86]. Therefore, it seems like a good idea to use phages in food safety which in turn come from food, especially for the consumer, who can identify fewer drawbacks than when consuming phages or their genetically manipulated enzybiotics.

Bacteriophages can also be used to prevent or to reduce colonization of domesticated livestock with bacterial pathogens before they enter the production chain [148]. After that, phages can be used to decontaminate inanimate surfaces made, for example, of stainless steel or to fight bacterial biofilms. Finally, phages can be used directly on food, both in unprocessed or ready-to-eat foods as well as processed foods, even stored at temperatures ranging from 4 °C to 20 °C.

Several cofactors tested with phages used in the control of *L. monocytogenes* in the food industry have been recently reviewed by Kawacka and coworkers [26,149]. Among those factors, we can find other bacterial cultures such as *Lactobacillus* spp., *Gluconocbacter assii*, the bacteriocins Nisin, Enterocin and Pediocin, and several compounds such as lauric arginate, potassium lactate, sodium diacetate, sucrose monolaurate.

## 3. Spatial Distribution of Phages

Bacteriophages’ ubiquity is another advantage. It is estimated that there are 10 bacteriophages for every bacterium present on our planet, representing a virtually unlimited source, not only of virions but also of lytic enzymes. Phages are especially abundant in seawater and soil and have also been found in large quantities in wastewater. The potential use of bacteriophages as indicators of environmental contamination has also been investigated in the last few decades [150,151,152,153,154,155]. Perhaps the most impressive figures are that phages kill bacteria at rates of up to 40% of the total population of marine bacteria per day and that carbon flux through phage biomass is estimated at 145 gigatonnes per year, playing a crucial role in our planet’s global carbon cycle [156,157]. They are also easily found on any animal or plant surfaces as they are part of the microbiota of most living things. Phages have also been isolated from a variety of foods, including ready-to-eat foods, fish and shellfish, milk products, meat, and vegetables [33,158,159,160,161,162]. Because of this, consumers are already in contact with food bacteriophages every day. Therefore, if researchers could offer an adequate explanation, it would help consumers to increase their acceptance of the use of food bacteriophages. In other words, they should accept their use as biopreservatives if we can explain well what this class of virus really is and how exactly they are used to fight “bad” bacteria in food.

## 4. Morphology and Classification

Initially, phages were characterized by transmission electron microscopy (TEM), followed by pulse-field gel electrophoresis and restriction endonuclease analysis. However, although TEM continues to be essential in publications on bacteriophage viruses, the quality of the images in many of the articles is questionable [163]. Most studies use the work of Ackermann or the criteria of the International Committee on Taxonomy of Viruses (ICTV) [164] to identify their phage isolates [165,166,167]. For further taxonomic classification and phage characterization, more detailed information, such as genomic data, has begun to be included in scientific publications [168,169,170,171].

Most phages belong to the order Caudovirales. Based on the tail morphology, Caudovirales are divided into three families: *Myoviridae*, *Siphoviridae*, and *Podoviridae*. *Myoviridae* phages are characterized by long straight contractile tails, *Siphoviridae* phages possess long flexible non-contractile tails, and *Podoviridae* phages have short, non-contractile tails [172].

Alternatively, we can also use the PCR technique and subsequent sequencing to partially characterize the isolated phages. For example, some authors used specific primers to detect the Major Capsid Protein (MCP) of reported *Salmonella* phages [158,159].

Augustine et al., also used PCR or multiplex PCR to perform a screening of virulence factors in DNA obtained from phages [35]. Tomat et al. used PCR to detect virulence factor genes (from diarrheagenic *E. coli* toxins) in two phages (DT1 and DT6) isolated from stool samples of patients with diarrhea [72].

**Figure 1:**
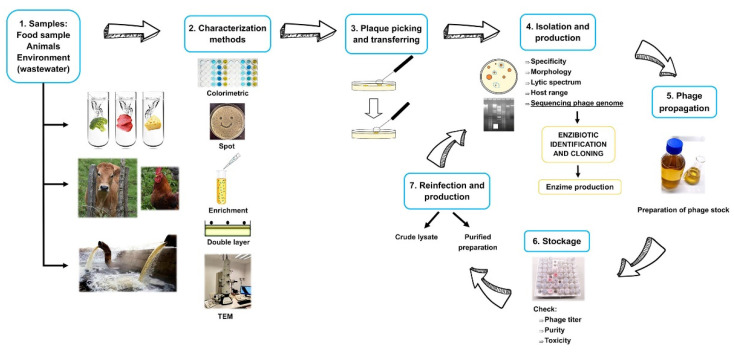
Steps followed for the isolation and characterization of phages.

Presently, full genome sequencing and analysis provide the key tool for taxonomic classification and for alerting the presence of “dangerous” genes that phage genomes may contain. We believe that it is necessary to sequence phage genomes to obtain information on the presence of antibiotic-resistant genes or virulence factors before determining their suitability for food applications. An outline with the steps followed for the isolation and characterization of phages for food biopreservation is shown in Figure 1.

DNA genomes of Caudovirales range in size from about 15 up to 500 kbp [173]. The study of the genome of phages is crucial today, but most investigations analyzed before to the last 10 years do not include the sequencing or annotation of these genomes. The complete genomes of phages are already included as a technique of characterization and phylogeny, but the in-depth analysis of these genomes has only been carried out very recently; this even allows us to discover new subfamilies and new genera of phages infecting food pathogens [43,125].

## 5. Phage’s Life Cycle

**Figure 2:**
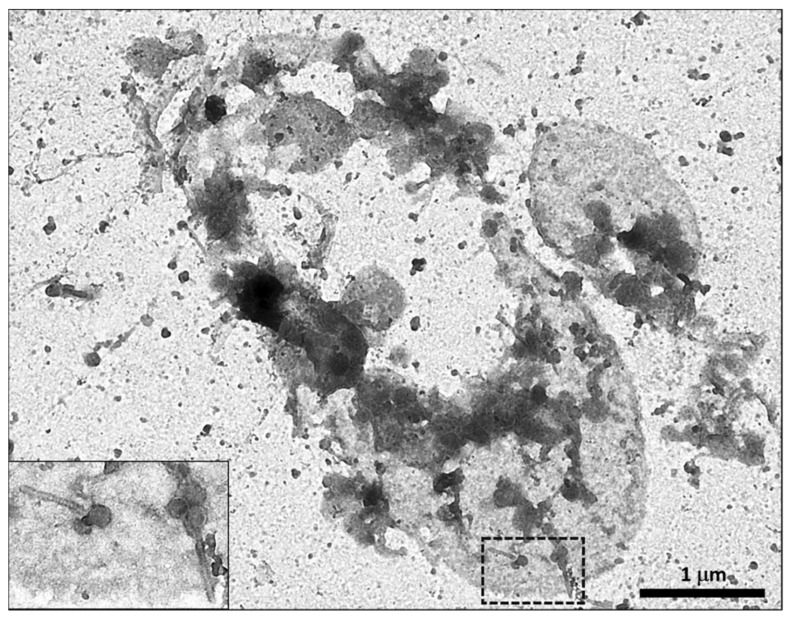
Gram-negative bacterium after lysis by phages. Numerous complete or incomplete phage heads and tails can be seen in the image. Inset: Detail of the boxed area showing two phages of the *Siphoviridae* family. Original magnification ×25,000.

To perpetuate themselves, phages must infect their host bacteria by binding to specific receptors on them. After injecting their nucleic acid into the bacterium’s cytoplasm, phages can hijack the bacterium’s cellular machinery to induce their own replication, through a process called the “lytic cycle”, giving rise to hundreds or thousands of complete viral particles that will leave the cell after killing it (Figure 2). Alternatively, if the phage nucleic acid is inserted into the chromosome or within a plasmid of the bacterium, it can remain in a kind of dormant state known as the “lysogenic cycle,” which will not produce new virus particles until conditions are favorable, or their genes are activated by some external stimulus. Lytic bacteriophages are the first choice to selectively kill bacteria in foods because lysogenic phages remain in the bacterial chromosome and will not multiply until the environment in which the bacterium is found allows for it, making lysogenic phages difficult to control.

## 6. Enzybiotics

There are three classes of bacterial cell wall hydrolases: animal lysozymes, bacterial autolysins, and phage lysins. All animal lysozymes share the ability to hydrolyze the β-(1,4)-glycosidic bond between the alternating N-acetylmuramic acid and N-acetylglucosamine residues of the bacterial cell wall polymer called peptidoglycan. Their biological role is mainly antibacterial defense, but some lysozymes also work as food digestive enzymes in animal guts [174]. Bacterial cell wall hydrolases are involved in carefully remodeling the cell wall to maintain cell integrity but also participate actively in processes such as cell division, bacterial surface appendages’ assembly, and the facilitation of bacterial secretion systems’ stabilization [175,176]. Most of these autolysins are peptidoglycan hydrolases (PGHs) that can provoke bacterial autolysis, so their expression and activity need to be tightly regulated.

The third class of cell wall hydrolases are phage endolysins, enzymes that directly target bonds in the peptidoglycan of the bacterial cell wall. These so-called enzybiotics (for ENZYme antiBIOTICS) are synthesized at the end of the bacteriophage lytic cycle to lyse the bacterium they parasitize, producing a lysis “from within” in Gram-negative bacteria [177]. Most endolysins contain one or two enzymatically active domains (EAD) in the N-terminus (which cleave one of the bonds in the bacterial peptidoglycan) and one cell wall-binding domain (CBD) in the C-terminal region (which is involved in host bacterial recognition). Based on their EAD, enzybiotics can be broadly divided into three types: endopeptidases, amidases, and glycosidases.

On the other hand, in Gram-positive bacteria, endolysins are also able to lyse bacteria “from outside” during the phage adsorption at the bacterial surface [178,179].

Endolysins have an extensive structural variation and a diverse cleavage predilection for the molecules with glycosidic, amide, or peptide bonds present in the bacterial peptidoglycan [180,181]. The structure of endolysins can be either globular or modular. Globular endolysins are unique for phages infecting Gram-negative bacteria, whereas modular endolysins are found in phages with a Gram-positive host. Another class of phage enzymes is virion-associated peptidoglycan hydrolases which share a similar mode of action on the bacterial peptidoglycan [182,183,184,185]. A good example of these newly studied antibacterial molecules is the virion-associated peptidoglycan hydrolase HydH5 of *Staphylococcus aureus* bacteriophage vB_SauS-phiIPLA88 [186]. Additionally, some phages can produce depolymerases to overcome bacterial protective layers such as proteinaceous S-layers [187] or polysaccharide capsules [188].

Among the advantages of enzybiotics, we include the possibility of totally or partially breaking the structure of bacterial biofilms. A biofilm can be defined as a structured community of bacterial cells enclosed in a self-produced polymeric matrix and adherent to an inert or living surface. Growth in biofilms enhances the survival of bacterial populations in the food industry environments, increasing the probability of causing food-borne infections. Due to the presence of extracellular material that protects biofilms, many phages have limited access to bacteria inside these structures. This can be solved using phages expressing exopolysaccharide depolymerases and endolysins. Endolysins can act effectively irrespective of the metabolic status of the cells (exponential and stationary phase cells) and are capable of killing planktonic cells as well as sessile cells. In this way, phage endolysins have been shown to be effective in eliminating biofilms formed by tenacious pathogens on different surfaces commonly used in the food industry [189,190,191,192]. Moreover, endolysins can be evaluated in combination with depolymerases or even with antibiotics to kill the underlying pathogen that formed the biofilm. On the other hand, as many pathogens build their biofilms based on different substances that form the biofilm matrix, it would be advisable to evaluate the activity of endolysins against biofilms that present a different proportion of proteins, nucleic acids, sugars or lipids.

Additionally, endolysins can kill “persister” bacteria that escape conventional antibiotics and even can kill the dreaded multi-resistant strains. Although there are not many studies in this regard, endolysins also offer the possibility of being used in combination with other molecules or with other solutions for the food industry, such as bacteriocins or probiotics. Furthermore, as gene-encoded proteins, enzybiotics are amenable to bioengineering strategies, both to optimize specificity and to increase yields [193,194]. An example is the construction of hybrid proteins consisting of LysSA11 -an endolysin of the *S. aureus* phage SA11 and the enzymatically active domain of LysB4- and endolysin from the *Bacillus cereus* phage B4 [195].

The search, characterization, and practical use of these phage-derived lysins have received less attention than phages, basically because they are more difficult for many laboratories to study. However, there is a growing body of work on these enzymes, particularly in the field of human and animal pathogens, which has encouraged researchers in other fields, including food safety, to begin promising work with enzybiotics. Not surprisingly, many enzybiotics have been successfully tested as biopreservatives or have been proposed by their discoverers as good candidates to be used in food against Gram-negative and Gram-positive bacteria (Table 2). The study of these enzymes in phages that do not belong to the “selective group” of food pathogens could provide a wide range of new proteins with different properties and varied spectra.

Furthermore, enzybiotics can improve the narrow host spectrum of phages against both Gram-positive and Gram-negative bacteria. Therefore, the narrow host range of phages should be used to control specific spoilage or pathogenic bacteria, while the broadest spectrum of enzybiotics can be used to control different strains or species. Some of the newly isolated and characterized endolysins have a broad spectrum so they could be candidates for use in the food industry. An example is endolysin M4Lys, which has a peculiar mosaic structure [222].

The main limitation in the use of phage enzybiotics in food is their complicated production and purification, since relatively large amounts of proteins are needed even to be studied in in vitro assays. Another problem is their low resistance to high temperatures used in different processes in the food industry, such as disinfection. However, the search for new enzymes with new properties will make it possible to find thermostable and easy-to-produce forms in heterologous hosts such as *E. coli* and *Lactococcus lactis* [21,221,223,224,225].

## 7. Concluding Remarks

Many natural and eco-friendly methodologies for food preservation have been proposed in the last few years, but only limited data are available about the usefulness of most of them under industrial scale conditions, which needs proper attention to satisfy the requirements of the industry as well as the demand of the consumers [226,227,228,229,230]. Consequently, studies about the ability of the reported biopreservative agents to control the development of undesirable microorganisms when applied at the industrial scale are greatly required.

Studies on the biocontrol of food-borne pathogens in foods have generally produced very good results. However, not all are lights in the use of phages against pathogenic bacteria in food, there are also shadows. There are assays in which it was not possible to reduce the number of pathogenic bacteria in food using bacteriophages [136,231,232].

The use of phages in human and veterinary medicine has received much more attention than their use in the food industry; but the increasing appearance of antibiotic-resistant strains in the food industry has begun to make these viruses be seriously taken into account when seeking their (application for food safety), also in this context. Similarly, their lytic enzymes have not been sufficiently exploited in the food industry to date. However, this is beginning to change; indeed, after the successful use of lysozyme (animal) or Nisin (bacteria), enzymes are beginning to be seriously valued in the food industry. Phages offer new and interesting possibilities when planning the control of annoying microorganisms in food manufacturing, food biopreservation, or food processing. Additionally, their lytic enzymes, easily modifiable through molecular biology processes, offer a very wide range of possibilities both for direct application against bacteria, as well as for inclusion in food matrices or the preparation of antibacterial surfaces generated by biotechnology [233].

Virulent bacteriophages are naturally present in foods, therefore both phages and their enzybiotics would be exploited in different ways for food safety as the consumer demand for the use of ecofriendly biopreservatives is increasing. Contamination of ready-to-eat products with pathogenic bacteria is a more serious problem than the contamination of food that will then be cooked before being consumed since many of the cooking methods reduce the number of these bacteria. In this context, both phage and enzybiotics have been tested in ready-to-eat meals. However, not only is the use of phages and their enzymes in food is not only an area of incipient research, but the whole biology of phages is experiencing a new boom in all domains of research, mainly in human and veterinary health, where spectacular achievements have already been reached in some patients and farm animals.

Along with this increasing amount of isolation and characterization of phage strains capable of controlling important food-borne pathogens—it is always desirable to increase our armament against superbugs—we must make a parallel effort to understand more in-depth their interaction with target pathogens, as well as their biology and ecology in food if we want to apply them in the different stages of the production chain, increasing their biopreservation capacity. At the molecular level, we must better characterize enzybiotics, study the possibility of applying them in different processes, and optimize their production so that their application is profitable for food producers and does not raise the price too much for consumers.

Furthermore, the safety and ubiquity of phages must be well explained to both food producers and consumers to avoid rejection of “the unknown” [234,235]. Bacteriophages are the most abundant microorganisms on the planet and even in our guts, with approximately 10^14^ phage particles in our body [236]. As we have seen in this review, phages and their enzybiotics can be found in the environment, in animals, and in food we eat every day. Finally, some phage-based products for the control of pathogens in food are already being used in different countries after being approved by competent authorities, even in ready-to-eat products. Those products mainly include a cocktail of phages, for example against *E. coli* (EcoShield™), *L. monocytogenes* (ListShield™ and PhageGuard Listex™), and *Salmonella* spp. (SalmoFresh™) [237].

## Figures and Tables

**Table 1:** Phages tested against food-borne pathogens and their proposed use as food biopreservatives.

Target Bacteria	Phage/s	Source	Characterization Method	Genome Length	^2^ Family	Food Application	Reference
*Aeromonas hydrophila*	AH-1	Sewage samples	TEM	ND	*Myoviridae*	Depuration of artificially contaminated cockles	[45]
	AH-4						
	AH-5						
*Bacillus cereus*	PBC1	Sewage sample	TEM, sequencing	41,164 bp	*Siphoviridae*	Inhibition of *B. cereus* growth in boiled rice	[46,47]
*Brochothrix thermosphacta*	A3	Spoiled retail rib steaks	TEM	ND	ND	Control of bacterial strains during refrigerated storage	[16,48]
*Campylobacter jejuni*	Cj6	Unknown	-	ND	ND	Control of pathogens in liquid foods	[36,49]
*C. jejuni*	2	Unknown	^1^ dsDNA	~140 kb	*Myoviridae*	Reduction of *C. jejuni* contamination of retail poultry products	[50,51]
*C. jejuni*	CP8 CP30	Poultry excreta	TEM, dsDNA	~140 kb	*Myoviridae*	Reduction of food-borne bacteria and biofilms	[52,53]
*C. jejuni*	12673	NCTC (UK)	TEM, DNA sequencing	~135 kb	*Myoviridae*	Reduction of bacterial contamination on chicken carcass surfaces	[54,55]
*Clostridium tyrobutyricum* and *C. sporogenes*	CTP1	Landfill	TEM, DNA sequencing	59,199 bp	*Siphoviridae*	Cheese manufacturing	[56]
*Cronobacter* (*Enterobacter*) *sakazakii*	ESP 1–3	Sewage treatment plant	TEM, dsDNA	ND	Siphoviridae	Control of *E. sakazakii* in reconstituted infant formula	[57]
	ESP 732–1				*Myoviridae*		
*Escherichia coli*	PE37	Bovine intestine samples	TEM, DNA sequencing	166,423 bp	*Myoviridae*	Biocontrol of *E. coli* STEC O157:H7 and ESBLEC.	[58]
*E. coli*	EC6	Sewage	TEM, dsDNA	ND	Siphoviridae	Biocontrol against *E. coli* in UHT and raw bovine milk	[59]
	EC9				*Myoviridae*		
	EC11				*Podoviridae*		
*E. coli* (STEC) O145	Ro145clw	Non-fecal compost samples	TEM, DNA sequencing	42,031 bp	*Siphoviridae*	Control of foodborne STEC O145	[60]
*E. coli* O157:H7	vB_EcoS_FFH_1	Wastewater treatment plants	TEM, sequencing	108,483 bp 139,020 bp	Siphoviridae	Reduction of contamination in ground beef	[61,62]
	_vB_EcoS_FFH_1				*Myoviridae*		
*E. coli* O157:H7	e11/2	Bovine farmyard Slurry samples Swine stool samples	TEM, dsDNA	ND ND ~140 kb	Myoviridae	Elimination or reduction of *E. coli* O157:H7 bacteria from meat carcasses	[63,64,65]
	e4/1c				*Siphoviridae*		
	PP01				*Myoviridae*		
*E. coli* O157:H7	FAHEc1	Raw screened sewage	TEM, dsDNA	~90 kb	*Myoviridae*	Inactivation of *E. coli* O157:H7 on beef	[34,66]
*E. coli* O157:H7	KH1	Cattle and sheep fecal samples	-	ND	ND	Elimination of O157:H7 from foods under refrigerated conditions. Reduction of *E. coli* on surfaces.	[67,68]
	KH4						
	KH5						
*E. coli* O157:H7	ECML-4	Fresh and salt water environments	TEM, DNA sequencing	157,308 bp 66,854 bp 166,783 bp	*Myoviridae*	Reduction of contamination of hard surfaces and foods contaminated by *E. coli* O157:H7	[69,70]
	ECML-117						
	ECML-134						
*E. coli* strains, *Salmonella* and *Shigella* spp.	C203	Cottage cheese and from poultry liver	TEM, DNA sequencing	138,073 bp	*Myoviridae*	Biocontrol agent against *E. coli* EHEC O157	[37]
	P206						
Shigatoxigenic *E. coli* Enteropathogenic *E. coli*	DT1	Stool samples of patients with diarrhea	TEM	ND	*Myoviridae*	Control of pathogenic *E.coli* in meat products and during milk fermentation	[71,72]
	DT5						
	DT6						
*E. coli* strains including serotype O157:H7	OSY-SP	Manure from cattle, sheep, and horse farms	Pulsed-field gel electrophoresis (PFGE)	~150 Kb	*Myoviridae*	Reduction of *E. coli* in fresh produce type (cut green pepper or spinach leaves)	[73]
*Lactobacillus brevis*	SA-C12	fresh silage	TEM	ND	*Myoviridae*	Control of *L. brevis* beer-spoilage	[74]
*Leuconostoc gelidum*	ggg	vacuum-packaged pork	TEM	ND	*Siphoviridae*	Inhibition of *Leuconostoc* in raw pork	[75]
*Listeria monocytogenes*	A500 ATCC^®^ 23074-B1™	*L. monocytogenes* isolated from Guinea pig	TEM	38,867 bp	*Siphoviridae*	Control of L-forms of *L. monocytogenes* on surfaces	[76,77]
*L. monocytogenes*	H387 H387-A 2671	-	TEM	ND	*Siphoviridae*	Disinfection of working surfaces of food processing plants	[78,79]
*L. monocytogenes*	LiMN4L	Seafood waste water treatment unit	ND	ND	ND	Control of *L. monocytogenes* on stainless steel in seafood processing environments	[22]
	LiMN4p						
	LiMN17						
*L. monocytogenes*	A511	Sewage from a sewage treatment plant	Phage typing, TEM, sequencing	134,494 bp	*Myoviridae*	Ready-to-eat foods from plant and animal origin including cheeses	[80,81,82,83,84]
*L. monocytogenes*	FWLLm1	Sheep feces	TEM,	ND	ND	Reduction of *L. monocytogenes* growth in ready-to-eat poultry products	[85]
*L. monocytogenes*	IZSAM-1	Floor drain-water from an Italian blue cheese dairy factory	TEM, sequencing	~50 kb	*Siphoviridae*	Biocontrol of *L. monocytogenes* within cheese industrial facilities	[86,87]
*Listeria* spp.	P100	Sewage effluent from a dairy plant	TEM, sequencing	131,384 bp	*Myoviridae*	Biocontrol of contaminated surfaces, the surface of soft cheeses, ready-to-eat foods, fresh-cut fruit, and fruit juices, raw fish fillets,	[88,89,90,91,92]
*Pseudomonas fragi*	Wy	Ground Beef	TEM, dsDNA	ND	-	Reduction of *P. fragi* in refrigerated raw milk	[93,94,95]
*Pseudomonas* sp.	C35	spoiled retail beef	-	ND	-	Biological control of beef spoilage	[96,97]
*Pseudomonas lactis*	HU1	sludge obtained after passing raw cow’s milk through a centrifugal clarifier	TEM, dsDNA	~48 Kb	*Podoviridae*	Control of *P. lactis* in Raw Cow’s Milk	[98]
*Pseudomonas fluorescens* *E. cloacae* strains	PspYZU5415	Sewage samples	TEM, sequencing	39,636 bp	*Siphoviridae Corticoviridae*	Growth inhibition of *E. cloacae* and *P. fluorescens* in cucumber juice with different salt concentrations	[43]
	EcpYZU01			39,767 bp			
*P. fluorescens*	IBB-PF7A	Sewage treatment plant	TEM, dsDNA	~42 kbp	*Podoviridae*	Biocontrol of *P. fluorescens* in dairy and other food industries	[99,100]
*Salmonella* Enteritidis, *S.* Typhimurium	wksl3	Chicken by-product samples	TEM, sequencing	42,766 bp	*Siphoviridae*	Control *Salmonella* on chicken skin. from broiler carcasses	[101]
*Salmonella* serovars	LPSEYT	Water samples	TEM, sequencing	53,387 bp	*Myoviridae*	Biocontrol of *Salmonella* in food matrices including milk and lettuce	[42]
*Salmonella* Enteritidis	CAU-SEP-1	River water samples	TEM	ND	*Myoviridae* and *Siphoviridae*	Control of *S. Enteritidis* in chicken breast meat	[102]
	CAU-SEP-2						
	CAU-SEP-3						
	CAU-SEP-4						
*Salmonella* Enteritidis	CNPSA 1 CNPSA3 CNPSA4	free-range chickens	TEM, dsDNA	ND	tailed dsDNA phages	Reduction of *Salmonella* Enteritidis in Contaminated Chicken Cuts	[103,104,105]
*Salmonella* Enteritidis	P29C	Raw human sewage	-	ND	*Siphoviridae*	Reduction of bacterial contamination on chicken carcass surfaces	[54,106]
*Salmonella* spp.	PSE5	Poultry slaughterhouse wastewater	plaque morphology and RAPD analysis	ND	ND	Reduction of contamination in raw chicken eggs	[107]
*Salmonella* spp.	LPSTLL	Environmentally water samples	TEM	ND	Siphoviridae	Reduction of *Salmonella* counts in milk and chicken breast and on stainless still surfaces	[108,109]
	LPST94				*Ackermannviridae*		
	LPST153				*Podoviridae*		
*Salmonella* strains	LPSE1	Environmental samples	TEM, dsDNA, sequencing	41,854 bp	*Siphoviridae*	Control of *Salmonella* in ready-to-eat foods	[110]
*Salmonella* strains	Felix O1/Felix O1-E2	Feces of paratyphoid B patients	TEM, Sequencing	86,155 bp/~84 kb	*Myoviridae*	Suppression of *Salmonella* growth on chicken frankfurters, poultry products, and ready-to-eat foods	[111,112,113,114]
*Salmonella* strains	PHL4	Wastewater treatment plant	-	ND	ND	Reduction of *Salmonella* growth poultry products	[115]
*Salmonella* strains	vB_SalS_SJ_3	Wastewater	TEM, DNA sequencing	162,910 bp	*Siphoviridae*	Biocontrol of *Salmonella* in contaminated Eggs and Pork	[116,117,118]
*Salmonella* strains	Pu20	Sewage samples	TEM, sequencing	59,435 bp	*Podoviridae*	Growth inhibition of *Salmonella* strains in liquid egg white and yolk	[119]
*Salmonella* strains	D1-2	Environmental samples	TEM, sequencing	86,878 bp	*Myoviridae*	Growth inhibition of *Salmonella* strains in liquid egg white and yolk	[120]
*Salmonella* Typhimurium	P22 [Argo4]	*Salmonella enterica* subsp. *enterica* serovar Typhimurium	TEM, sequencing. Reference strain ATCC^®^ 97540™	41,724 bp	*Podoviridae*	Prevention of attachment to food surfaces and food matrices	[121,122,123,124]
*Salmonella* Typhimurium	P7	Unknown	-	ND	ND	Control of pathogens in liquid foods	[36]
*Salmonella* serovars	LPST153	Water samples	TEM, sequencing	39,176 bp	*Autographivirinae*	Control of *Salmonella* in raw milk and raw beef sausages	[125]
*S. enterica* serovar Typhimurium *S. enterica* serovar Enteritidis	UAB_Phi 20	Chicken	TEM, dsDNA, sequencing	41,809 bp 44,110 bp 87,669 bp	*Podoviridae* *Podoviridae Myoviridae*	Reduction of *Salmonella* on foods and reduction of *Salmonella* Colonization of poultry	[126,127,128]
	UAB_Phi78	Chicken					
	UAB_Phi87	pig					
*Salmonella* Enteritidis	SP-1	Intestinal content of broiler chickens	TEM, dsDNA, PCR amplification	~86 kb	Podoviridae	Biocontrol of *Salmonella* in cooked chicken meat	[35,129,130]
	SP-3			~88 kb	*Siphoviridae*		
*Salmonella* Enteritidis	SJ2	Chicken egg	ND	ND	ND	Reduction of *Salmonella* counts in Cheddar cheese made from both raw and pasteurized milk, and in contaminated eggs and pork	[131]
*Salmonella* Enteritidis	vBSenM-PA13076 (PA13076) vBSenM-PC2184 (PC2184)	Chicken sewage	TEM	52,474 bp ND	*Myoviridae*	Biocontrol of *Salmonella* in foods (chicken breast, pasteurized whole milk, Chinese cabbage)	[132,133]
*Salmonella* and *E. coli* O157:H7	PS5	Raw chicken products	TEM, sequencing	158,400 bp	*Myoviridae*	Reduction of viable counts on solid and liquid foods	[134]
*Salmonella Oranienburg*	SSP5 SSP6	Sewage samples	TEM	ND	*Myoviridae* *Siphoviridae*	Control of *Salmonella* Oranienburg on alfalfa seeds	[135]
*S. Typhimurium* *S. Enteritidis* *S. Montevideo*	A B	sewage treatment plant	TEM	ND	*Myoviridae Siphoviridae*	Control of *Salmonella* in mustard and broccoli seeds	[136]
*Salmonella* strains, including MDR *Salmonella*	T156	Waste water	TEM, dsDNA, sequencing	123,849 bp	*Siphoviridae*	Microencapsulated bacteriophage applied in skim milk and lettuce for biocontrol of *Salmonella*	[137]
*Staphylococcus aureus*	K	Deposited by EA Asheshov	ATCC^®^ 19685-B1™	139,831 bp	*Myoviridae*	Removing *S. aureus* biofilms	[138,139]
*S. aureus*	H5 (phiPLA88) A72 (phiPLA35)	Raw milk	TEM, dsDNA, sequencing	42,526 bp 45,344 bp	*Siphoviridae*	Curd manufacturing, fresh and hard-type cheeses	[140,141,142]
*S. aureus*	SA46-CTH2	Food samples	TEM	17,505 bp	*Podoviridae*	Inactivation of *S. aureus* planktonic cells in pasteurized milk and biofilms on stainless steel surfaces	[143]
*S. aureus*	SA13m	Temperate phage SA13 isolated from a goat fecal sample	TEM, sequencing	42,652 bp	*Siphoviridae*	Biocontrol of *S. aureus* in pasteurized whole milk at refrigeration and ambient temperatures	[144]
*Shewanella baltica* and *S. putrefaciens*	SppYZU01 to SppYZU10	Wastewater from freshwater and marine product marketplaces	TEM, sequencing	SppYZU01 (43,567 bp) SppYZU05 (54,319 bp)	*Myoviridae Siphoviridae*	Biopreservation of chilled channel catfish	[145]
*Shigella* spp.	SF-A2	Spiced chicken	TEM	ND	*Myoviridae*	inactivation of foodborne *Shigella* on ready-to-eat chicken	[146]
	SD-11	Pig farm effluent					
	SS-92	Pig farm effluent					
*Vibrio parahaemolyticus*	vB_VpaS_OMN (designated as phage OMN)	Sea water	TEM, sequencing	42,202 bp	*Podoviridae*	Inactivation of *V. parahaemolyticus* in oyster meat	[147]

^1^ Nuclease digestion tests and/or Random Amplified Polymorphic DNA Analyses (RAPD), ^2^ Family designated by the authors, ND: not determined, TEM: Transmission Electron Microscopy.

**Table 2:** Enzybiotics tested against food-borne pathogens and their proposed use in foods.

Target Bacteria	Enzybiotic	Source	Food Application	Reference
*Bacillus cereus*, *B. subtilis* and *L. monocytogenes*	LysB4	*B. cereus* phage B4	antibacterial agent to control foodborne pathogens.	[196]
*Clostridium tyrobutyricum* and *C. sporogenes*	Ctp1L	Bacteriophage CTP1 isolated from landfill	Cheese manufacture, reduction of clostridial activity in cheese	[56,197]
*C. tyrobutyricum* *C. acetobutylicum*	CS74L	Lytic bacteriophage (ATCC^®^ 8074-B1^TM^) of *C. sporogenes*	Biocontrol of clostridia strains in foods	[198]
*C*. *perfringens*	Ply3626	*C. perfringens* ATCC 3626	Control of anaerobic spore-formers	[199]
*C. perfringens*	LysCPAS15	*C. perfringens* phage CPAS-15	Inhibition of *C. perfringens* in sterilized milk	[200]
*Bacillus subtilis* *B. megaterium* *L. monocytogenes*	PLY118, PLY500 PLY 511	Phages from *Listeria monocytogenes*	Production of airy starter cultures with biopreservation properties	[201,202,203]
*E. coli* O157:H7	PlyEc2	Phage from *E. coli*	Reduction of *E. coli* O157:H7 on contaminated lettuce	[204]
*Lactococcus lactis, Pediococcus acidilactici* and *P. pentosaceus*	LysA2	*L. casei* bacteriophage A2	Ripening of fermented products	[205]
Lactobacilli, lactococci, pediococci, *B. Subtilis* *Brevibacterium linens* *Enterococcus faecium*	Mur-LH	Phage 0303 from *Lactobacillus helveticus* CNRZ 303	Preventing the growth of spoilage microbes	[17]
*L. monocytogenes* *B. subtilis*	PlyP100	Phage from *L. monocytogenes*	Antimicrobial biopreservative in fresh cheese.	[206]
*L. monocytogenes*	LysZ5	Phage from *L. monocytogenes*	Control pathogens in soya milk	[207]
*L. monocytogenes*	PlyLM	Phage from *L. monocytogenes strain 4b*	Proposed control of *L. monocytogenes* in food matrices and processing facilities	[208]
*L. monocytogenes*	HPL118 HPL500 HPL511 HPLP35	Recombinant endolysins from *L. monocytogenes* phages	Reduction of *L. monocytogenes* viable counts in iceberg lettuce. Promising perspectives in production and packaging environments	[201,209,210]
Methicillin-resistant *Staphylococcus aureus*	LysGH15	Phage isolated from Sewage samples	Biopreservative in whole and skim milk	[211,212]
methicillin-resistant *S. aureus*	LysSA11	*Staphylococcus aureus* phage SA11	Biocontrol of *S. aureus* on strain in pasteurized milk or ham and utensils	[213]
*S. aureus* *Bacillus cereus*	Hybrid LysB4EAD-LysSA11	Phage SA11 from *S. aureus* phage B4 S from *B. cereus*	Biocontrol of *S. aureus* and *B. cereus* in boiled rice	[195]
*S. aureus*	LysH5	Staphylococcal bacteriophage phi-SauS-IPLA88	Disinfection process of industrial food facilities. Elimination of *S. aureus* in pasteurized milk	[190,214]
*S. aureus*	CHAPSH3b	Chimeric protein (CHAP domain from peptidoglycan hydrolase HydH5 and the SH3b cell wall-binding domain from lysostaphin)	*S. aureus* biofilm elimination	[215]
*S. aureus*	CHAP_K_	Truncated derivative of the phage lysin LysK from the staphylococcal bacteriophage K	Reduction of biofilm formation in processing systems	[189]
*S. aureus*	HydH5 HydH5Lyso HydH5SH3b CHAPSH3b and lysostaphin	*S. aureus* bacteriophage vB_SauS-phiIPLA88	Biocontrol of *S. aureus* in dairy products	[216]
*Streptococcus* spp.	λSA2	*Streptococcus agalactiae* (serotype III GBS strain 3330) bacteriophage B30	Inactivation of *Strepcococcus* spp. in cow milk	[217,218]
*S.* Typhimurium	LysSTG2	*Salmonella*-lytic bacteriophage STG2	Combating *S.* Typhimurium biofilms in food industries	[219]
*Salmonella strains*	LysSE24	*Salmonella* phage LPSE1	Food Control of *Salmonella* strains	[220]
Several Gram-negative pathogens, particularly against *Salmonella* Typhimurium	Lys68	*Salmonella* phage phi68 isolated from feces from a poultry farm	Combat Gram-negative pathogens in the food industry	[221]
